# Gastrointestinal Myoelectrical Activity (GIMA) Biomarker for Noninvasive Diagnosis of Endometriosis

**DOI:** 10.3390/jcm13102866

**Published:** 2024-05-13

**Authors:** Mark Noar, John Mathias, Ajit Kolatkar

**Affiliations:** 1 Endometriosis and Neuroenterology Research Institute, 53 Loveton Circle, Sparks Glencoe, MD 21152, USA; 2 Woman’s Hospital of Texas, 7600 Fannin Street, Houston, TX 77054, USA; jmat479309@gmail.com; 3 Gastrointestinal Consultants of Houston, 7501 Fannin St., Suite 705, Houston, TX 77054, USA; 4 Specialty Business Center, Integrative Ayurveda Gastroenterology and Nutrition Initiative, 202, Balewadi, Pune 411045, Maharashtra, India; dr.ajit@gastrolab.com

**Keywords:** biomarker, electroviscerography, non-invasive electroviscerography, endometriosis, gastrointestinal myoelectrical activity (GIMA), electroviscerogram, water load satiety test, GIMA biomarker threshold score, predictive modeling

## Abstract

**Background/Objectives:** Endometriosis represents substantial direct and indirect healthcare costs impacted by an absence of uniformly accurate, non-invasive diagnostic tools. We endeavored to demonstrate gastrointestinal myoelectrical activity (GIMA) biomarkers, unique to endometriosis, will allow non-invasive, uniformly accurate diagnosis or exclusion of endometriosis. **Methods:** Prospective open-label comparative study of 154 patients, age ≥ 18, with or without diagnosed endometriosis. Population included 62 non-endometriosis controls (Cohort 1), 43 subjects with surgically/histologically confirmed endometriosis (Cohort 2), and 49 subjects with abdominal pain and negative imaging (Cohort 3). Non-invasive electroviscerography (EVG) recorded GIMA biomarkers from three abdominal electrodes before and 30 min post water load protocol. Cohort 2 had postoperative EVG and Cohort 3 had preoperative EVG. Calculated specificity, sensitivity, negative predictive value (NPV), positive predictive value (PPV), and predictive probability or C-statistic used univariate, multivariate, linear, and logistical regression analyses of the area under the curve (AUC) at all frequency and time points, including age and pain covariants. **Results:** The non-endometriosis cohort differed significantly from the endometriosis cohorts (*p* < 0.001) for median (IQR) and AUC percent frequency distribution of power at baseline, 10 min, 20 min, and 30 min post water load at all frequency ranges: 15–20 cpm, 30–40 cpm, and 40–50 cpm. The endometriosis cohorts were statistically similar (*p* > 0.05). GIMA biomarker threshold scoring demonstrated 95%/91% sensitivity and PPV, 96%/95% specificity and NPV, and a C-statistic of >99%/98%, respectively, for age subsets. GIMA biomarkers in Cohort 3 predicted 47/49 subjects positive and 2/49 negative for endometriosis, confirmed surgically. Hormonal therapy, surgical stage, nor pain score affected diagnostic accuracy. **Conclusions:** EVG with GIMA biomarker detection distinguished participants with and without endometriosis based upon endometriosis-specific GIMA biomarkers threshold scoring.

## 1. Introduction

Endometriosis is a chronic, complex estrogen-driven disorder, with genetic and immunologic-driven variation where endometrial tissue is found in extra-uterine sites. The disease elicits local and systemic inflammation, fibrosis, and pain [1,2,3]. It affects 6–10% of premenopausal women and teens, 60% of those with chronic pelvic pain, 80% of patients with dysmenorrhea, and 30–50% of women with infertility [2]. The disease prevalence is likely underestimated, and misdiagnosis is common due to a lack of both patient and healthcare provider training and awareness. Other factors include the normalization of dysmenorrhea symptoms, especially in teens, as well as cultural mores around menstruation and pain in women, and symptoms that are not specific to the disease in its many presentations [1,2]. The reported accuracy of the current diagnostic standard of laparoscopic surgery and histopathology is only 50% to 75%, which is problematic [4]. Women see multiple practitioners over 8–12 years until correctly diagnosed, often hampered by unintended geographic and financial factors [5]. As a result of these factors, there is an average of 8.6 years from the time of symptom appearance, until the time of final diagnosis, during which the disease continues to advance. This exacts an immeasurable toll on the quality of life while disrupting educational and career goals, as well as personal relationships [5,6]. The economic impact is underrecognized with US direct medical costs of USD 26 billion and lost productivity costs of USD 55 billion annually [7].

The long-term risks of untreated endometriosis, including infertility, depression, and links to other chronic diseases, such as ovarian cancer, cardiovascular disease, and autoimmune diseases [7], is driving the need for new methods and technologies for early diagnosis. Diagnostic tests like MRI, or, magnetic resonance imaging, and transvaginal ultrasound are highly accurate but only in more advanced disease which represents only 15–20% of symptomatic women. However, the diagnostic accuracy is lost after surgical intervention leaving no options for the post-treatment evaluation of disease [4]. The remaining 70% of women suffer for an average of 8.5 years before diagnosis can be made due to a lack of low-cost, accurate, non-invasive, and readily available diagnostic testing. Current non-invasive testing is resource-intensive and hampered by variable diagnostic accuracy in addition to requiring the acquisition, storage, transport, complex analysis, and disposal of biological materials [8,9,10,11,12,13]. Questions regarding reproducibility and genetic or ethnic variability have not yet been fully addressed [14]. New diagnostic testing shows great promise and awaits multicenter randomized control trials for further validation and broader applications; for example, assessing disease and symptom recurrence across the lifespan [14].

Concerns regarding the accuracy and expense of surgery, the current diagnostic standard, have led to guideline changes, suggesting the use of non-invasive technology, such as MRI and ultrasound as a diagnostic equivalent [15]. The publication of the #Enzian classification for the non-invasive characterization of endometriosis further emphasizes the need for validated non-invasive, cost-effective, and accurate diagnostic testing [16]. This is even more imperative when considering that in a study of 2017 people with endometriosis from 63 countries, patients experienced an average delay of 3.7 years between symptom onset and first presentation of symptoms to a physician (the care-seeking delay) and an average delay of 5.8 years between first presentation of symptoms to a physician and diagnosis of endometriosis (the healthcare-related delay), with an average total diagnostic delay of 9.6 years seeing more than 4–5 providers. This was commonly attributed to being ignored because they were considered unreliable, and participant character attributes (e.g., age, appearance, and weight or physical ability) leading to clinician dismissal [17].

### 1.1. State of Diagnostic Testing

During the past several years, a number of diagnostic tools have evolved and been presented as promising in the setting of endometriosis. These have consisted of blood- and saliva-borne mRNA fragments, blood-borne mutated DNA [18], endometrial brushings looking at cellular irregularities or chemical parameters such as BCL6 [19], menstrual fluid analysis for levels of uterine killer cells [20], or levels of CXCL5 and IL1RN [21]. 

The introduction of diagnostic biomarkers, such as salivary mRNA [10] and others which could fulfill the need, has not resulted in a change in the current recommendations to use biomarkers [22]. This has been attributed to reported low accuracy, or in some cases, a lack of more extensive validation. 

In a prior published study, using transnasal placed motility catheters for 24 h, a unique small bowel motility pattern was observed. This represented a biomarker of gastrointestinal myoelectrical activity (GIMA) showing a unique range of contractile frequencies specific to the diagnosis of endometriosis [23]. Subsequent evaluation of the GIMA biomarker using EVG technology confirmed the original study findings and demonstrated an unexpected 100% C-statistic, sensitivity, and specificity from the running spectral analysis display of the GIMA biomarker in an initial small trial demonstration cohort [24].

### 1.2. Physiological Concept of Disease Response

Over 31 different cytokines are produced by the female reproductive system, as are large quantities of prostaglandin E2 (PGE2), F-alpha (PGF-a), and I2 (PGI2), with a half-life of ≤30 s [25]. PGF-a causes simultaneous contraction of longitudinal and circular smooth muscle resulting in spasm. PGE2 promotes peristalsis in the small bowel, fallopian tubes, and uterus, essential for egg transport, initiating menstruation, and delivery. Endometriosis is associated with elevated PGE2 and PGF-a secretion in peritoneal explants, fluid, and serum [26,27,28], with resultant non-propulsive small bowel motility, seizure-like activity, and high-frequency bowel patterns [23]. PGE2 and PGF-a, not normally produced in simultaneously elevated quantities except by endometriosis tissue, disables small bowel smooth muscle motor control resulting in high-frequency spasm, detected as GIMA biomarkers. No other diseases are known to produce simultaneously elevated PGE2 and PGF-a. The effect occurs in a drug dose–response curve manner. Studies of over 500 subjects with other gynecological, urological, and gastrointestinal diseases failed to demonstrate endometriosis-associated GIMA biomarkers (Noar-Unpublished Data-10 February 2023). 

### 1.3. Study Basis

The initial robust results of the GIMA biomarker technology to diagnose endometriosis compelled the design of the current multicenter, multi-ethnic study of the novel GIMA biomarker with AI-derived threshold scoring, using EVG technology, to validate: (1) the diagnostic accuracy of the unique signature of endometriosis utilizing the GIMA biomarker fingerprint, (2) the ability of the test to distinguish between subjects with and without disease, and (3) additional validation of the AI algorithm threshold model based upon the number of variables and the number of patients required to satisfy performance thresholds to maintain high diagnostic accuracy. The current data represents the results of this validation trial of the EVG-detected GIMA biomarker in subjects with and without endometriosis. 

## 2. Materials and Methods

### 2.1. Study Design and Disease Overview

#### 2.1.1. Ethics Statement

The study information and data used in the analysis were obtained as part of a prospective study protocol which was reviewed and approved by the human investigational review board IRB—C.H.C.A. Woman’s Hospital, L.P., 00004260, Houston, TX, USA, for a non-randomized open-label prospective comparative study to investigate the detection of a novel gastrointestinal myoelectrical activity biomarker for endometriosis by comparing participants with known and suspected endometriosis, healthy asymptomatic women, and subjects with abdominal/pelvic symptoms from other diseases without a diagnosis of endometriosis. Informed consent was obtained from all subjects as a condition to study inclusion. STARD reporting guidelines were observed for study and data analysis [29].

#### 2.1.2. Study Population and Sample Size

The full study population was composed of 165 women, aged 18 or older. Subjects were recruited from a woman’s specialty clinic and gastroenterology practice into one of three cohorts depending on if the criteria for inclusion was satisfied: Cohort 1 consisted of asymptomatic subjects without signs or symptoms of endometriosis, or participants with other documented disease-associated abdominal pain not diagnosed as endometriosis. Cohort 2 consisted of subjects with histologically documented endometriosis by biopsy or excisional biopsy, but which did not undergo total excision at laparoscopy. There was no segmental resection of bowel, bladder, or vagina, which would have resulted in exclusion from the study. Cohort 3 included women complaining of abdominal or pelvic discomfort, with negative diagnostic testing, including ultrasound, transvaginal/transrectal ultrasound, or MRI, who were suspected to have endometriosis and scheduled for diagnostic laparoscopy. Participating clinicians were blinded to EVG and/or surgical results and EVG technicians were blinded to surgical results. Subjects found to have endometriosis had an assigned stage based upon the revised American Society for Reproductive Medicine classification (rASRM) [30]. No changes were made to existing treatment modalities, including birth control medication, medicated IUD’s, other hormonal therapy, or GnRH modulators.

Exclusion criteria included: ASA physical status classification ≥ III, gastrointestinal tumor or ulcers, stenosis or mechanical bowel or urinary obstruction, prior gastrectomy, extensive small bowel resection or pelvic surgery, or malignancy.

Based upon the prior pilot study, an exact test using a linear multiple regression random model and a single tail was used to estimate the sample size required to reach a particular threshold of performance with a low alpha error probability of 0.05 and a 95% confidence level, with 2 predictors. A sample size of 44 subjects allowed for an actual power of 95%.

#### 2.1.3. Study Procedures and Protocol

Participants underwent complete history and physical examinations, completed a standardized pain questionnaire, and underwent EVG (3CPM Company, Inc., Sparks Glencoe, MD, USA, Software Version 2.09i) with a water load satiety test (WLST). Participants who satisfied exclusion and inclusion criteria were stratified into one of three main cohorts. Non-endometriosis Cohort 1, subgroup 1A, asymptomatic subjects without signs or symptoms of endometriosis, and subgroup 1B, participants with other documented disease-associated abdominal pain not diagnosed as endometriosis. Endometriosis Cohort 2 had histologically documented endometriosis without total excision at laparoscopy before EVG testing, and endometriosis Cohort 3 included participants having abdominal or pelvic discomfort, suspected to have endometriosis and scheduled for planned laparoscopy, after EVG testing.

#### 2.1.4. Electroviscerogram with a WLST

**Figure 1:**
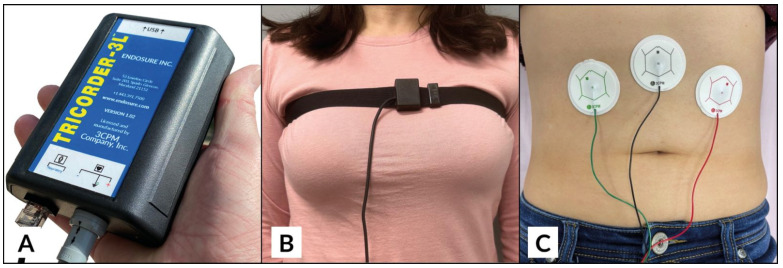
EVG system with (**A**): tricorder-3l, (**B**): respiratory belt, and (**C**): dry gel electrodes.

Following an overnight fast of 6–8 h, subjects underwent a standardized EVG (3CPM Company, Inc., Sparks Glencoe, MD, USA, Software Version 2.09i) with a WLST study. Subjects were placed in a 30° to 45° reclining position, wearing loose-fitting clothing. Following standard protocol, three dry gel electrode pads were applied to the anterior abdomen halfway between the umbilicus and xiphoid process, and 5 cm below the costal border at the midclavicular line on both left and right sides. A respiratory sensor belt was placed across the upper chest to distinguish respirations from bowel activity (Figure 1). Initial equipment and calibration testing was conducted over a 3–5 min period of time after which the subject remained quietly in the same position for a baseline study, lasting 10 min.

At the end of the 10 min baseline period, the subject began a standardized water load stimulation. The purpose of the WLST was to cause gastric distention with activation of the gastric pacemaker and subsequent activation of small bowel contractility. This is considered an essential part of any gastrointestinal motility study. The WLST consisted of drinking room-temperature water until the subject indicated that they felt completely full. The WLST usually took approximately 2 to 5 min, with the subject typically ingesting between 300 to 1000 cc of fluid. The amount ingested was recorded. The ingestion of the fluid took place in the same position as during the baseline and for the remainder of the study. After the WLST, the subject remained motionless, in a reclining position for a 30 minute period of time at which point the study was completed. Results were then immediately available.

The standardized EVGs were recorded using an FDA-cleared hand-held EVG device and respiratory belt to distinguish respirations from bowel contractions [23,24,31]. Three silver chloride electrodes were positioned on the abdomen. EVGSAS custom software version 2.09i (3CPM Company, Sparks Glencoe, MD, USA) was used to perform recording and data analysis of measurements of filtered percent distribution of power at 15–20, 20–30, 30–40, 40–50, and 50–60 cpm ranges during baseline recording and 10, 20, and 30 min after water load. These are the GIMA biomarker frequency ranges specific to endometriosis [23,24].

Additionally, a running spectral analysis (RSA) was created, stratifying frequency over time and AUC measurements at specified frequency ranges to provide visual recognition of disease-state GIMA biomarker abnormal frequencies versus normal range values. The RSA is a visual representation of biomarker activity over time and provides a visual diagnosis of disease. However, for more precise statistical analysis, it is the AUC that is used. The percentage frequency distribution of power of the AUC was used to determine sensitivity, specificity, positive and negative predictive values as well as the diagnostic predictability.

#### 2.1.5. Pain/Discomfort Score

Pain was calculated using a modified ENDOPAIN 4D standardized pain questionnaire [32]. Participants used a 10-point verbal rating scale for categorizing the pain associated with menstruation, urination, sexual intercourse, defecation, and otherwise general levels of abdominal and pelvic pain. The calculated score was the highest single score of reported items.

#### 2.1.6. Statistical Methods 

Baseline characteristics of Cohorts 1–3 were compared using a rank-sum test and categorical variables using Fisher’s exact test. Baseline GIMA biomarker characteristics of Cohort 1 Subgroups 1A and 1B were assessed for similarity.

GIMA biomarkers were measured at baseline, 10–, 20–, and 30–min for frequencies 10–60 cpm. Unadjusted median and inter-quartile range (IQR) were calculated for each combination of time and frequency and compared within Cohort 1 by presence and absence of symptoms and across cohorts using a rank-sum test. Box plots were used to demonstrate the difference between cohorts for the distribution of frequencies 10.0–60.0 cpm at each time.

A univariable and multivariable mixed-effects linear regression model assessed differences over time by cohort separately per biomarker frequency. Multivariable models were adjusted for age, BMI, symptom score, and water load quantity. AUC for frequencies 10.0–60.0 used trapezoidal rule calculation. Median AUC per frequency was compared between cases and controls by a rank-sum test. Kernel density plots were used to assess and exhibit AUC differences between the cohorts. Distribution of AUC by cohorts was explored using a box plot. Univariable and adjusted mean AUC differences by cohorts were estimated using linear regression models. Univariable and adjusted mean AUC differences by cohorts were estimated using linear regression models. Multivariable models were adjusted for age, BMI, symptom score, and water load using logistic regression analysis for GIMA biomarker predictive modeling and calculating sensitivity, specificity, negative and positive predictive values, and the C-statistic of the diagnostic test. The cut-off level for significance used to conduct the statistical analysis for the study was 5%. All statistical analyses utilized R 4.2.2.

## 3. Results

### 3.1. Description of the GIMA Biomarker Cohorts 

**Figure 2:**
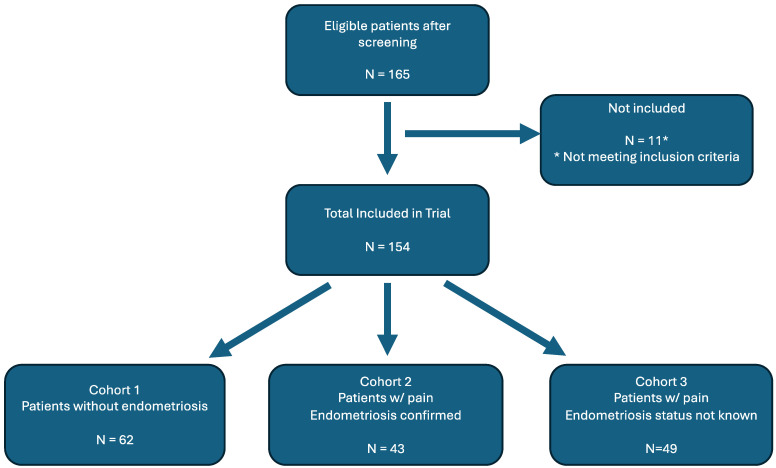
Cohort stratification.

There were 165 subjects initially recruited into the study, of which 65 were in Cohort 1, 50 were in Cohort 2, and 50 in Cohort 3. Three of the subjects in Cohort 1, seven from Cohort 2, and one from Cohort 3 did not meet the inclusion criteria due to failure to obtain informed consent or complete pain scores (Figure 2).

All remaining subjects were able to successfully undergo an EVG with a WLST after completing pain questionnaires and informed consent. It was possible to obtain adequate running spectral analyses and sufficient data to be able to calculate the AUC. Artificial intelligence-derived GIMA threshold scores were calculated for each subject using the AUC data at 30–40 cpm and 40–50 cpm at time points 10–, 20–, and 30–min post water load. 

The clinical and demographic characteristics of the 154 patients in the study are presented in Table 1 and Table 2. With regard to age, non-endometriosis Cohort 1 differed significantly from both endometriosis-positive Cohorts 2 and 3. The slightly younger age of the two endometriosis-positive Cohorts 2 and 3 could affect the power of the study. However, the strength and uniformity of the data when comparing the non-endometriosis versus the endometriosis-positive cohorts did not suggest any influence caused by the age difference. Ethnicity did not differ significantly in the endometriosis-positive Cohort 3 vs. non-endometriosis Cohort 1 (*p* = 0.49) but statistical differences were seen between Cohort 1 and 2 (*p* < 0.001) and endometriosis-positive Cohort 2 and 3 (*p* = 0.04). Women were more likely to be Asian (0% vs. 21%) and less likely to Caucasian (82% vs. 67%) when comparing Cohort 1 and 2. Women were more likely to be Asian in Cohort 2 (21%) but more likely to be Caucasian (85%) in Cohort 3. BMI did not differ in the three cohorts and was statistically similar. An expected statistically significant (*p* < 0.001) difference was observed in symptom scores between the healthy controls and EM-positive Cohort 2 and 3 (*p* < 0.001). But no statistical difference was observed between Cohort 2 and 3 (*p* = 0.07). Pain was highly associated with EM-positive cohorts, with a statistically significant difference (*p* < 0.001) observed between EM-negative Cohort 1 and EM-positive Cohorts 2 and 3. Abdominal pain was reported by 89% Cohort 3 participants and 98% in Cohort 2 versus 69% in Cohort 1. Bloating was noted in 70% and 60% of Cohorts 3 and 2, respectively, compared to 45% in Cohort 1. ENDO-4D pain scores, used a VAS ranging from 0–10, with 10 being the highest level of pain, rating the pain score using median IQR (Table 1).

Clinical conditions and comorbidities, medication use, and surgical staging results are shown in Table 2.

There were 154 subjects in the study. A total of 90 had histologically documented endometriosis, with 43 in endometriosis-positive Cohort 2 and 47 in endometriosis-positive validation Cohort 3. The originally recruited 62 subjects not documented as having endometriosis were divided between 25 asymptomatic and 37 symptomatic non-endometriosis controls. An additional 2 subjects were subsequently noted to be negative for endometriosis in the validation Cohort 3 after trial entry and planned laparoscopy. Of those with endometriosis, ASRM stages 1–4 were reported to be represented equally, without significant difference between staging.

Comorbidities in the non-endometriosis control group were diabetes and insulin sensitivity in 5/62 (8%), constipation/IBS 9/62 (15%), and PCOS, fibroids, simple ovarian cysts, inflammatory bowel disease, collagen vascular disease, interstitial cystitis in the 2–5/62 or 3–8% range. The absence of comorbidities in endometriosis-positive Cohorts 2 and 3 were not significant (60% vs. 65%) but were significantly higher than the non-endometriosis Cohort 1 of 53%.

Hormonal therapy was noted in 16 (26%) of the non-endometriosis Cohort 1 and 24 (56%) of the endometriosis-positive Cohort 2, and 36 (64%) of Cohort 3. Cohort 1 hormonal treatment consisted of 9 (14%) combined oral contraceptive pill, 4 (6%) estrogen only, 2 (3%) progestin, 1 (2%) medicated IUD, and no GnRH agonist, while for subjects in Cohort 2, 11 (26%) reported oral contraceptive pill, 3 (7%) progestin, 1 (2%) androgen, 1 (2%) medicated IUD, 2 (5%) estrogen only, and 6 (14%) GnRH agonist. Cohort 3 hormonal treatment consisted of 14 (29%) combined oral contraceptive pill, 6 (12%) progestin, 3 (6%) GnRH agonist, 7 (14%) estrogen only, and no medicated IUD.

### 3.2. RSA—Qualitative Analysis—GIMA Biomarker Fingerprint Pattern Recognition

**Figure 3:**
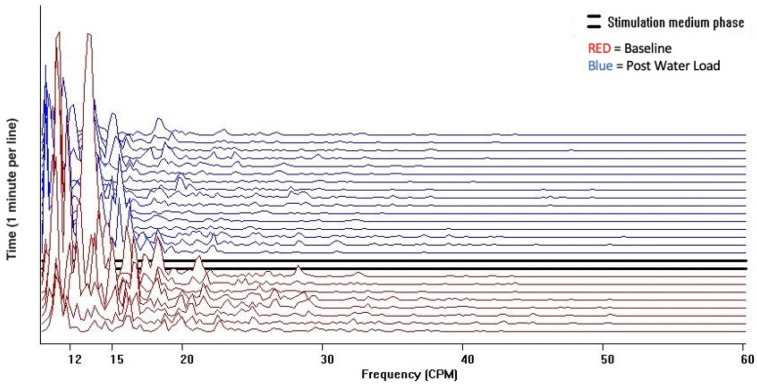
Running spectral analysis of Cohort 1. Power of frequency distribution of GIMA characteristics at frequencies (10–60 cpm) among non-endometriosis participants, who were with or without symptoms at baseline and 10 min, 20 min, and 30 min post water load.

The Cohort 1 qualitative visual pattern was flat both at baseline and after water load, without the unique diagnostic 15–60 cpm GIMA biomarker pattern. (Figure 3).

**Figure 4:**
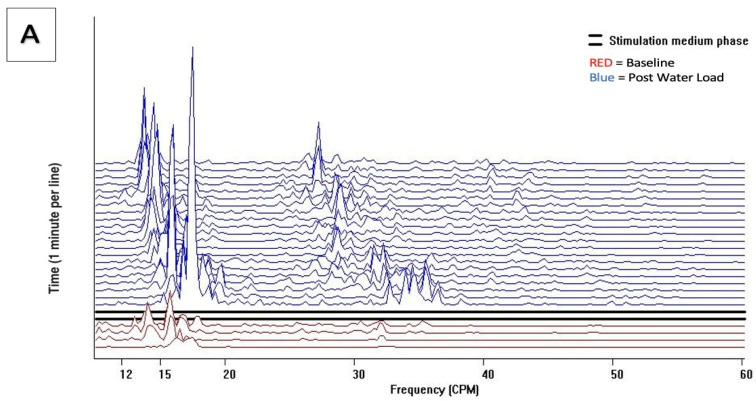

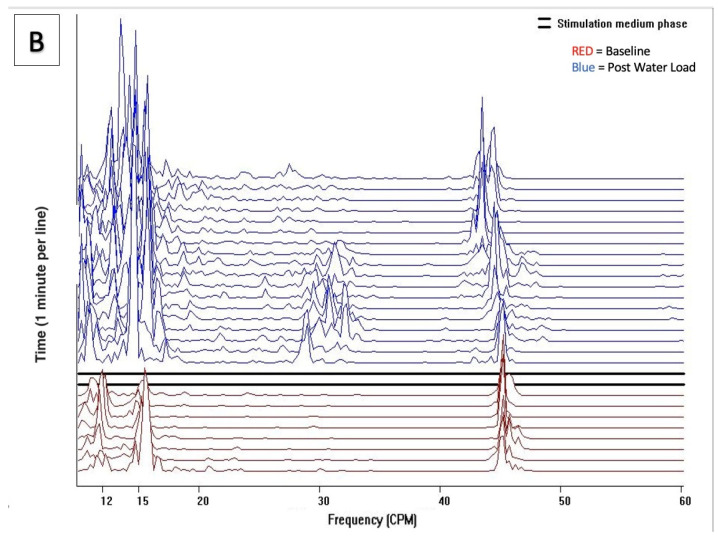
Running spectral analysis of (**A**) Cohort 2 and (**B**) Cohort 3. Power of frequency distribution of GIMA characteristics at frequencies (10–60 cpm) among subjects with endometriosis, Cohort 2 and Cohort 3, at Baseline and 10 min, 20 min and 30 min post water load.

Comparatively, endometriosis-positive Cohorts 2 and 3 demonstrated significant and visually distinct patterns with increased activity in both the baseline and post water load periods with increased GIMA biomarker activity in the 15–20 cpm, 30–40 cpm, and 40–50 cpm frequency ranges representing known endometriosis-associated GIMA biomarker activity (Figure 4A,B). These differences were noted in 43/43 of Cohort 2 subjects, 47/49 of Cohort 3 subjects, but were absent in all 62 non-endometriosis Cohort 1 subjects as well as absent in 2/49 (4%) of Cohort 3 subjects who did not have endometriosis at the time of surgery. These qualitative findings were confirmed by histopathological positivity and/or absence of endometriosis.

### 3.3. EVG GIMA Biomarker Predictive Modeling

**Figure 5:**
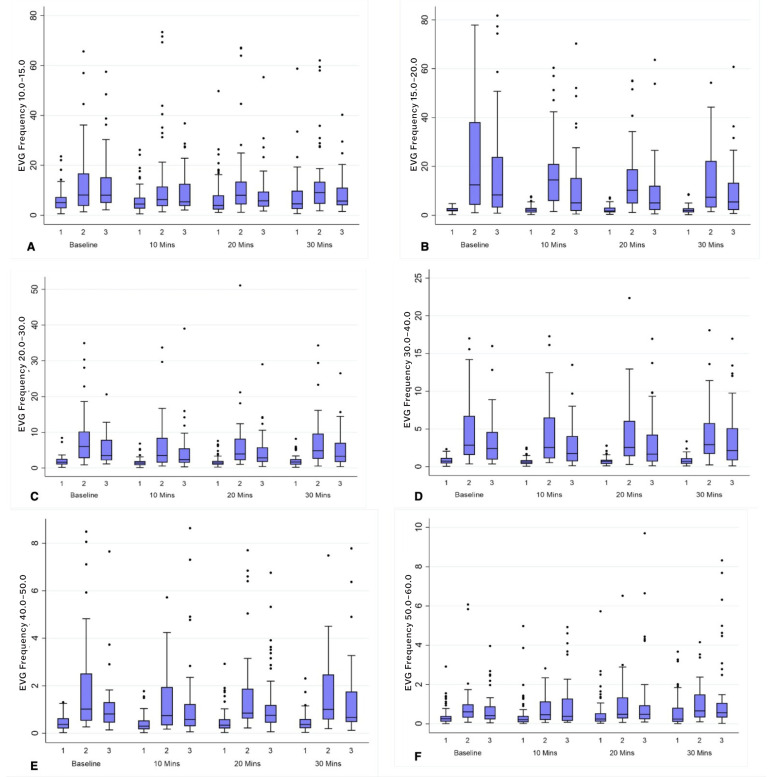
Box plots, panels A-F, depict comparison of distribution of GIMA characteristics using median (IQR). Unadjusted median and interquartile ranges for frequencies (10–60 cpm) among healthy controls (Cohort 1) versus subjects with endometriosis (Cohorts 2 and 3) at BL, 10 min, 20 min and 30 min. (**A**) = EVG Frequencies 10–15 cpm, (**B**) = EVG Frequencies 15–20 cpm, (**C**) = EVG Frequencies 20–30 cpm, (**D**) = EVG Frequencies 30–40 cpm, (**E**) = EVG Frequencies 40–50 cpm, and (**F**) = EVG Frequencies 50–60 cpm).

The comparison of EVG GIMA biomarkers was derived as the percent frequency distribution of power for median (IQR) between non-endometriosis controls (Cohort 1) versus endometriosis-positive women (Cohort 2 and 3). Frequencies of 15–60 cpm at baseline, 10–, 20–, and 30–min after water load were found to be significantly different (*p* < 0.05) as seen in Table 3 and Figure 5. Cohort 2 and Cohort 3 were similar for all frequencies and time combinations except for 15–20 cpm at 10 min and 20 min (Table 3). The GIMA biomarker-positive endometriosis participants in Cohort 3 had the same quantitative GIMA biomarker findings as histologically positive endometriosis Cohort 2 subjects.

**Figure 6:**
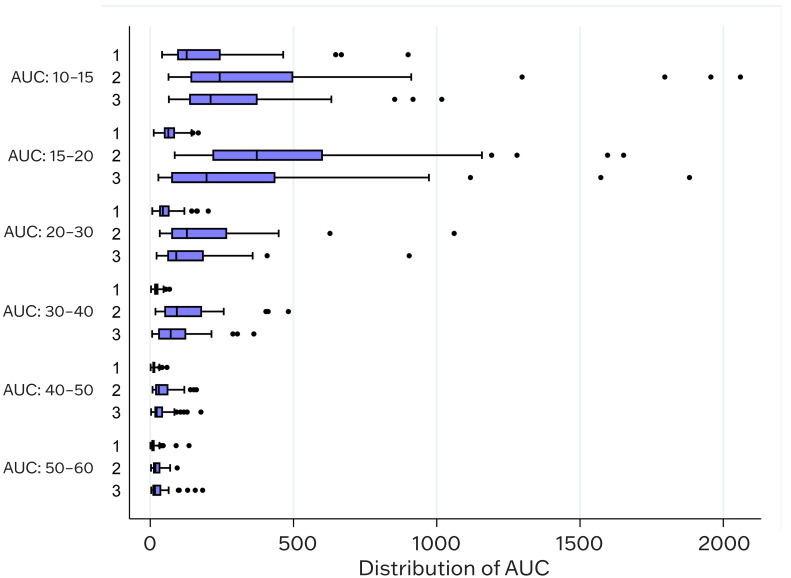
Distribution of AUC for frequencies between healthy, non-endometriosis controls (Cohort 1) and subjects with endometriosis (Cohorts 2 and 3). Box plots were used to compare distribution of AUC between controls and cases for a given frequency. For all frequencies, *p* value was less than 0.001 while comparing Cohort 1 and Cohort 2 and Cohort 1 and Cohort 3 from 15 cpm to 60 cpm.

Moreover, AUC values of the area under the RSA curves calculated for all frequency cycles was significantly higher (*p* < 0.001) for Cohort 2 (Table 4a), as well as for Cohort 3, (Table 4b), with the exception of the AUC frequency difference of 10–15 cpm, which is still significantly higher at a *p* = 0.003 versus non-endometriosis Cohort 1 (Figure 6 and Figure S1A–F).

AUC value of the area under the RSA curves for higher frequencies were similar between both endometriosis-positive cohorts (Cohort 2 and 3) with the exception being for frequencies 15–20 cpm and 30–40 cpm which demonstrated a significant difference (*p =* 0.005 and *p* = 0.04, respectively) (Table 4c).

Additionally, linear regression analysis of AUC for differences between non-endometriosis controls (Cohort 1) and women with endometriosis (Cohort 2 and Cohort 3) was significant (*p* < 0.001) at 15–20 cpm, 30–40 cpm, and 40–50 cpm frequency ranges (Table 5).

**Figure 7:**
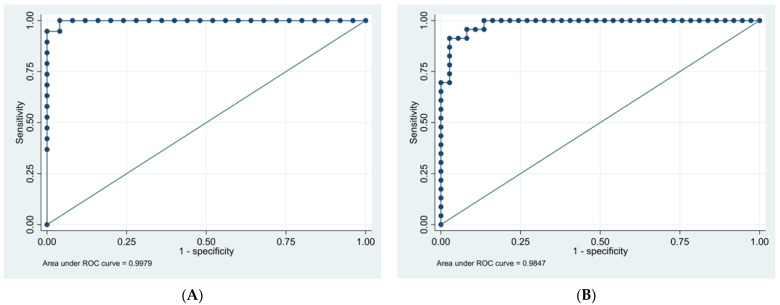
(**A**). ROC curve for disease predictability age ≤ 35 years. Area under the ROC curve of GIMA biomarker AUC is 0.9979. (**B**). ROC curve for disease predictability age ≥ 36 years. Area under the ROC curve of GIMA biomarker AUC is 0.9847. The closer to a value of 1.0, the higher the predictive value of the test.

More specifically, the ROC of the GIMA biomarker AUC graphs (Figure 7A,B) affirm that women with a higher GIMA biomarker AUC are more likely to have a diagnosis of endometriosis, correlating with the high specificity and predictability attributed to the GIMA biomarker. Additionally, based upon the AI-derived analysis which included the variables age and pain score in addition to the AUC, an appreciable difference between women 35 and younger and 36 and older was noted. This is seen in the differences in disease predictability in the ROC of the AUC curves in Figure 7A,B.

As previously demonstrated in Table 4a, EVG identifies not only participants with endometriosis, but also those without the disease despite other concomitant illnesses present. This is evidenced by the absence of the distinct fingerprint or GIMA biomarker of endometriosis in both Cohort 1 subgroups.

Notably, the non-endometriosis control group, Cohort 1, was made up of women without symptoms (n = 7) as well as subjects with pain-causing symptoms due to other concomitant and potentially confounding illnesses (n = 55). For this reason, the GIMA biomarker characteristics of each subgroup were compared to establish statistical homogeneity of the non-endometriosis control group. Both subgroups were noted to be substantially statistically similar (Table 6). Despite the disparity in numbers of the subgroups, the significant homogeneity of the analysis establishes the validity of the comparison. The data validate the GIMA biomarker as a means to non-invasively predict or exclude endometriosis in asymptomatic or symptomatic women with other concomitant illness.

### 3.4. EVG Ai Derived GIMA Biomarker Algorithm for Predicting Endometriosis

Area under the curve (AUC) was calculated for each GIMA frequency per participant measured at baseline, 10–, 20–, and 30–min. Multivariable logistic regression models were used to assess the effect of AUC GIMA frequencies and confounding variables age and symptom score. Analysis was stratified by participants older than 35, or 35 and younger, as explained previously. Permutations of AUC frequencies were used in the model (Table 7 and Table 8). AI methods revealed the most parsimonious model with the highest C-statistic values and lowest rate of misclassification to estimate the probability of disease.

Notable is the purposeful decision to exclude the 15–20 cpm frequency AUC from the final ideal AI-derived calculation, due to the previously noted statistically significant difference between Cohorts 2 and 3 at that frequency level. This is most likely accounted for by the presence of gallbladder disease disproportionally noted between Cohorts 2 and 3 (see Table 2). Gallbladder disease is known to induce duodenal wall spasm in the 12–20 cpm range [33] Therefore, elimination of this frequency band avoids the potential to have gallbladder disease as a confounding factor in the diagnosis of endometriosis.

Predicted probability of endometriosis was calculated from the logistic regression equation. Women were classified as having endometriosis if the predicted probability was >50%. This supervised predictive model diagnosed endometriosis if the estimated probability was higher or equal to the threshold value of ≥0.5 and excluded disease if the estimated probability was less than the threshold value of <0.5.

### 3.5. GIMA Biomarker Model Performance

Based upon the AI-derived analysis which included the variables age and pain score in addition to AUC, an appreciable difference between women 35 and younger and 36 and older was noted. Therefore, the data of each age-determined subgroup were calculated separately to avoid age-related bias.

In endometriosis-positive Cohort 2, the model displayed a 95% sensitivity, 96% specificity, 95% positive predictive value (PPV), 96% negative predictive value (NPV), and a >99% C-statistic for women ≤ 35 years.

In women ≥ 36 years, the model displayed 91% sensitivity, 95% specificity, 91% PPV, 95% NPV, and a 98% C-statistic, with a drop of 4% in sensitivity and PPV (Table 7 and Table 8).

When applied to the endometriosis-positive validation cohort (Cohort 3) to confirm the proportion of women who could be identified correctly using the prediction model, 91% were correctly classified, with a 91% C-statistic and 96% specificity noted. (Table 9) Variance from Cohort 2 is accounted for by the previously noted variance in the AUC _15–20_ and AUC _30–40_ between Cohorts 2 and 3 (Table 4c). In addition, in endometriosis-positive Cohort 3 there were two subjects that were predicted and found to be negative for endometriosis. Rather than excluding these subjects, their data were included in the Cohort 3 data pool, resulting in a slight decline in model prediction and the AUC variance in the 15–20 cpm and 30–40 cpm ranges.

## 4. Discussion

At this time, this analysis of GIMA biomarkers in subjects both with endometriosis, and without endometriosis, with and without other non-endometriosis pain-associated disease, appears to be the first prospective study to report a unique GIMA biomarker signature or fingerprint for endometriosis. The impact of early diagnosis, in the current healthcare environment, of an accurate, non-invasive test for endometriosis with immediate results and not requiring the usual infrastructural time and costs of current testing methods would be profound. The combination of a non-invasive EVG device which detects the novel GIMA biomarker and AI-derived diagnostic threshold modeling represents its intrinsic value. In a disease known for complexity and lack of predictability and homogeneity, the stability of this mature technology married with modern analytical methodology and a uniform, unique myoelectrical reaction in response to endometriosis provides accuracy and reproducibility.

Implicit to the development of the EVG system to take advantage of the GIMA biomarker association with endometriosis was that it would result in a low-cost, resource-efficient system that could be easily deployed by anyone, anywhere in the world where there was access to a computing device, water to drink, and a place to recline. The overriding goal was to eliminate the unintended financial or geographic discrimination of delayed diagnosis, stemming from current bottlenecks such as facility/provider access, infrastructural costs of laboratory-based testing, transport and processing, pressure on costly resources such as room and personnel time, and equipment availability.

In the diverse milieu of endometriosis, this prospective multicenter validation trial confirms the diagnostic capabilities as well as the accuracy and performance of the non-invasive EVG technology detection of the GIMA biomarker unique to endometriosis. The stability and reproducibility of the GIMA biomarker coupled with the AI-derived diagnostic threshold level for detecting endometriosis and distinguishing the disease from non-disease status is confirmed by the data. In addition, the accuracy regardless of ethnicity suggests potential applicability across international populations. The two centers where the study was performed represent two different medical models spanning from the expert tertiary care center to the routine outpatient setting, with differing levels of severity of disease presentation.

The study demonstrated the ability to detect and differentiate between endometriosis-confirmed subjects and non-endometriosis controls, with 95% sensitivity and PPV, 96% specificity and NPV, and 99% and 98% C-statistic predictability in women ≤ 35 years old and ≥36 years old women, respectively. The results are predicated upon GIMA biomarker activity driven by PGE2- and PGF-a-mediated 15–60 cpm smooth muscle GIMA activity, unique to endometriosis. Variations related to age, concurrent hormonal therapy, or stage of the disease had no impact, nor did the presence of confounding illnesses like inflammatory bowel diseases, irritable bowel syndrome, urinary or pelvic infection, chronic interstitial cystitis, biliary or ulcer disease, and polycystic ovary disease. The results of this larger formal prospective study, with a validation cohort, compared favorably and validated the results of an earlier pilot study. In the initial pilot study, the EVG technology was able to use the GIMA biomarkers to accurately distinguish between subjects with and without surgically confirmed endometriosis [24].

The application of this technology will likely be influenced by the disease severity. Advanced stage disease noted in 15% of women is easily diagnosed with transvaginal or transanal ultrasound, MRI, or physical exam [9]. In the advanced stage subgroup, GIMA biomarker detection will be useful in providing long-sought-after post-therapeutic monitoring, currently limited by diagnostic accuracy and differentiation between postoperative changes and recurrent disease. The most compelling subgroup is the over 70% of women with early-stage disease who remain symptomatic and suffer from lack of diagnosis or inadequate response to medical therapy. GIMA biomarker threshold scoring could reduce delayed diagnosis of endometriosis of those remaining undiagnosed for decades with an average of 8.3 years between symptom onset and diagnosis in patients with pain, contrasted with 1.8 years in those with infertility [34,35]. Cultural complexities, sexual discrimination, and geographic and financial barriers influence delays [6].

In the current medical environment, non-invasive diagnostic options are either lacking, expensive, or have limited availability or diagnostic accuracy. Ref. [8] Transvaginal ultrasound and MRI are effective but limited in early disease or post-surgical treatment. [9] Blood mRNA or mutated DNA elements have varied accuracy, requiring biological material handling and laboratory services [10,11]. Menstrual fluid and endometrial scrapings face similar challenges [12,13]. The more recent use of salivary mRNA shows promise and although there is reported sensitivity and specificity in the 95% to 100% range, AUC diagnostic accuracy is variable, ranging from 69–98%, with further challenges due to logistics and high cost [10,36,37]. In contrast, GIMA biomarker testing does not require the logistical challenges of biological sampling, laboratory infrastructure, specialized facilities, and cost.

As with any diagnostic test to be introduced into routine clinical practice, significant external validation will be required to answer the necessary questions concerning specificity and applicability across broad, diverse ethnic populations with variable disease. However, once accomplished, immediate clinical benefits of timely, non-invasive diagnosis are earlier access to therapy for symptom control and limitation of disease progression. No guarantees exist regarding disease advancement, and randomized clinical trial data, with laparoscopy before and after an interval without treatment, demonstrated 30% short-interval advancement, without a means of predicting progression [38].

Low-cost, accurate, and mobile testing would decentralize access, allowing universal rapid deployment, shortening the time between initial presentation, diagnosis, and treatment. With reproducibility in general populations, a positive impact on participants, caregivers, medical costs, and productivity is expected, eliminating unintended geographical or financial discrimination [39].

Further positive impacts include improved (1) understanding of the natural history of the disease and differences/similarities between ethnic groups or countries, (2) post-surgical or medical treatment monitoring and detection of recurrence, (3) new medication development, and (4) improved adolescent dysmenorrhea screening. Threshold scores of similarly derived biomarkers may allow differential identification of other presentations of endometriosis, adenomyosis, autoimmune diseases, or malignancies like ovarian cancer with unique diagnostic GIMA biomarkers. Employment of GIMA biomarker threshold scoring may play an important decisional role especially in the 47–71% of patients that have no evidence of residual endometriosis after undergoing repeat surgery following complete excision of all recognized endometriosis [40,41]. It may limit unnecessary repeat surgeries and additionally help to refocus on finding either unsuspected palpable nodules missed laparoscopically, or non-visualized unrecognized bowel endometriotic satellites [42].

### Strengths and Limitations

The EVG device resembles devices including the handheld electrocardiogram, making it intuitive, recognizable, and practical [24,31] with procedural proficiency after 1–2 procedures. Low procedural costs as well as the minimal technical proficiency required to perform the test will translate into greater availability.

At this stage, inherent limitations need to be recognized. These types of studies performed in ideal or tertiary research settings may not translate directly into real-world clinical practice and further testing is needed in larger varied populations of patients. Secondly, as in all studies including a control group not suspected of having endometriosis, finding ideal disease-free control groups is inherently difficult with many ways to miss endometriosis [41] even in asymptomatic women and with a recognized surgical diagnostic accuracy of only 50–75% [4]. In the control group of this analysis, it was encouraging to see the homogeneity of absent GIMA biomarkers compared to those with known disease.

Moreover, while the data are statistically sufficient to justify conclusions, model-building is an evolving process. Continued experience will refine the model, account for potential population variances, improve predictability, confirm GIMA biomarker threshold score variations, predict surgical stage/disease activity, facilitate post-treatment monitoring, and assess hormonal suppression impact. Hormonal suppression did not affect study results, yet newer suppressants may exert unknown effects. Other unidentified coexisting diseases may simultaneously secrete PGE2 and PGF-a, presenting as false positive results. The potential that adenomyosis could have similar GIMA biomarkers requires further evaluation of the possible impact on GIMA biomarker threshold scoring. Finally, it is not yet possible to make broadly definitive conclusions regarding the potential confounding effects of ethnic variation or other inflammatory diseases, which will require study in larger affected patient cohorts.

## 5. Conclusions

An unmet need exists for cost-effective, widely available, accurate, non-invasive endometriosis testing. This analysis of a prospective multicenter study provides data on the diagnostic accuracy of the non-invasive EVG detection of the unique GIMA biomarker to distinguish between subjects with or without disease. Further marriage of the EVG biomarker detection with AI-derived threshold scoring has demonstrated a non-invasive tool with beyond reasonable accuracy to diagnose endometriosis. Testing predicted endometriosis with 98%–99+% accuracy regardless of surgical stage or hormonal therapy. With further validation in larger study cohorts and other confirmatory studies, it is reasonable to expect that this new, non-invasive test and others will permit timelier diagnosis of this devastating disease, and may lead to: (1) discovery of additional biomarkers for other benign and malignant disease, (2) improved out of control costs of direct care and lost productivity, (3) increased accuracy of return to surgery decisions, (4) pretesting prior to routine surgery, (5) prenatal screening to detect unexpected endometriosis which is associated with significant post-partum complications [43], and (6) entry of new therapeutics into the marketplace.

## 6. Patents

The following patents are related to the work reported in this manuscript:

- (1) U.S. Patent No. 7,160,254. Intelligent Self-interpreting Electroviscerogram System and Method.
- (2) U.S. Patent No. 11/369,310. Method and System for Predicting Successful Treatment Methods and Outcomes of Bodily Tissue Disorders Based On Energy Activity of the Tissue.

## Figures and Tables

**Table 1:** Comparison of baseline demographic characteristics in non-endometriosis Cohort 1 and endometriosis-positive Cohorts 2 and 3.

Baseline Characteristics	Cohort 1 N = 62	Cohort 2 N = 43	Cohort 3 N = 49	Cohort 2 vs. Cohort 1	Cohort 3 vs. Cohort 1	Cohort 3 vs. Cohort 2
Age, Median (IQR)	40 (30–49)	32 (27–38)	36 (27–38)	0.003	0.01	0.42
BMI, Median (IQR)	25.1 (20.6–29.1)	24.4 (21.1–28.6)	23.6 (19.9–27.4)	0.90	0.18	0.14
Ethnicity, n (%)						
Asian Black Caucasian Hispanic	0 3 (5%) 51 (82%) 8 (13%)	9 (21%) 2 (5%) 29 (67%) 3 (7%)	1 (2%) 3 (7%) 39 (85%) 3 (7%)	0.001	0.49	0.04
ENDO-4D Pain Score, Median (IQR) range 0–10	1.5 (0–3.0)	4.0 (3.0–5.5)	5.0 (4.0–6.0)	*p* < 0.001	*p* < 0.001	0.07
Pain						
No Yes	19 (31%) 43 (69%)	1 (2%) 42 (98%)	5 (11%) 42 (89%)	*p* < 0.001	0.02	0.62
Bloating						
No Yes	34 (55%) 28 (45%)	17 (40%) 26 (60%)	14 (30%) 33 (70%)	0.17	0.01	0.34

Medians were compared using a rank-sum test and percentages were compared using a Fischer’s exact test. A *p*-value of less than 0.05 was considered to be statistically significant.

**Table 2:** Clinical characteristics of the GIMA biomarker cohorts.

Characteristic	Cohort 1 Non-Endometriosis Patients (*n* = 62)	Cohort 2 Endometriosis Surgically Confirmed (*n* = 43)	Cohort 3 Post-Surgical Confirmation Endometriosis (*n* = 49)
Mode of diagnosis			
Surgically Confirmed	-	43/43	47/49
Surgically Excluded	-	0	2/49
ASRM Classification			
I–II	-	23/43	25/47
III–IV	-	20/43	22/47
MEDICATIONS, *n* (%)	16 (26%)	24 (56%)	30 (64%)
Oral Dual Contraceptive	9 (14%)	11 (26%)	14 (29%)
Progestins	2 (3%)	3 (7%)	6 (12%)
Androgens	0	1 (2%)	0
Medicated IUD	1 (2%)	1 (2%)	0
GnRH agents	0	6 (14%)	3 (6%)
Estrogen only	4 (6%)	2 (5%)	7 (14%)
Control diagnoses (not endometriosis) *n* (%)			
No abnormality	33 (53%)	26 (60%)	32 (65%)
Polycythemia vera	1 (2%)	0	0
Microscopic/Ulcerative Colitis/Crohn’s	3 (5%)	0	0
Ehlers Danlos	1 (2%)	0	0
Thyroid Disease	2 (3%)	1 (2%)	2 (4%)
PCOS	2 (3%)	1 (2%)	1 (2%)
Collagen Vascular Disease	3 (5%)	3 (7%)	2 (4%)
Interstitial Cystitis	3 (5%)	4 (9%)	3 (6%)
Fibroids	5 (8%)	3 (7%)	1 (2%)
Simple Ovarian Cysts	2 (3%)	2 (5%)	4 (8%)
IBS	9 (15%)	3 (7%)	3 (6%)
Diabetes	5 (8%)	4 (9%)	7 (14%)
Migraines	1 (2%)	1 (2%)	0
Gallbladder Disease	0	2 (5%)	0

**Table 3:** Comparison of GIMA biomarker characteristics using median (IQR) in non-endometriosis Cohort 1 and endometriosis-positive Cohorts 2 and 3.

Frequency (Cycles/Min)	Cohort 1 (n = 62)	Cohort 2 (n = 43)	Cohort 3 (n = 49)
10.0–15.0			
Baseline	5.1 (2.8–7.3)	8.1 (3.8–16.7)	8.1 (5.0–15.2) *
10 min	4.5 (2.7–7.1)	6.3 (3.7–11.4)	5.4 (3.7–12.6) *
20 min	4.0 (2.3–8.1)	8.0 (4.4–13.5)	5.8 (3.5–9.5) *
30 min	4.6 (2.5–9.9)	9.1 (4.6–13.4)	5.7 (4.0–11.1) *^,^**
15.0–20.0			
Baseline	2.1 (1.6–3.0)	12.4 (4.3–38.1)	8.4 (3.2–23.8) *
10 min	2.1 (1.1–3.0)	14.4 (5.8–21.0)	5.1 (1.8–15.2)
20 min	1.7 (1.2–3.1)	10.3 (4.9–18.8)	5.0 (2.2–12.0)
30 min	2.0 (1.2–2.8)	7.4 (3.2–22.2)	5.4 (2.2–13.3) *
20.0–30.0			
Baseline	1.6 (1.0–2.6)	6.0 (2.8–10.2)	3.5 (2.1–7.9) *
10 min	1.4 (0.8–1.9)	3.5 (1.5–8.4)	2.4 (1.5–5.5) *
20 min	1.4 (0.8–1.9)	3.9 (2.2–8.2)	2.8 (1.8–5.8) *
30 min	1.7 (0.9–2.5)	4.8 (2.6–9.6)	3.3 (1.7–7.0) *
30.0–40.0			
Baseline	0.7 (0.4–1.2)	2.9 1.6–6.7)	2.4 (1.0–4.6) *
10 min	0.6 (0.4–0.9)	2.5 (1.1–6.5)	1.7 (0.7–4.0) *
20 min	0.6 (0.4–0.9)	2.6 (1.4–6.1)	1.7 (0.7–4.3) *
30 min	0.7 (0.4–1.2)	2.9 (1.7–5.8)	2.1 (0.9–5.1) *
40.0–50.0			
Baseline	0.4 (0.2–0.6)	1.0 (0.5–2.5)	0.8 (0.4–1.3) *
10 min	0.3 (0.2–0.5)	0.7 (0.3–1.9)	0.6 (0.3–1.2) *
20 min	0.3 (0.2–0.6)	0.8 (0.6–1.9)	0.8 (0.6–1.2) *
30 min	0.4 (0.2–0.6)	1.0 (0.6–2.5)	0.7 (0.5–1.8) *
50.0–60.0			
Baseline	0.3 (0.1–0.4)	0.6 (0.3–1.0)	0.4 (0.2–0.9) *
10 min	0.2 (0.1–0.4)	0.5 (0.2–1.1)	0.4 (0.2–1.3) *
20 min	0.2 (0.1–0.5)	0.5 (0.3–1.3)	0.5 (0.2–0.9) *
30 min	0.2 (0.1–0.8)	0.7 (0.3–1.5)	0.6 (0.3–1.1) *

Unadjusted median and interquartile ranges calculated for each frequency ranging from 10 cpm to 60 cpm at times BL, 10, 20, and 30 min comparing non-endometriosis controls (Cohort 1) and subjects with endometriosis (Cohort 2 and Cohort 3) using rank-sum test. *p* value of less than 0.05 is statistically significant. * *p*-value > 0.05 for comparing Cohort 2 and Cohort 3; ** *p*-value > 0.05 for comparing Cohort 1 and Cohort 3. All other *p*-values are <0.05.

**Table 4:** (**a**). Summary of GIMA biomarker AUC taken from area under the RSA curve of non-endometriosis controls (Cohort 1) and subjects with endometriosis (Cohort 2). (**b**). Summary of GIMA biomarker AUC taken from area under the RSA curve of non-endometriosis controls (Cohort 1) and subjects with endometriosis (Cohort 3). (**c**) Summary of GIMA biomarker AUC taken from area under the RSA curve of endometriosis-positive Cohort 2 and Cohort 3.

(**a**)			
**AUC Frequency**	**Cohort 1**	**Cohort 2**	***p*-Value**
10–15 cpm	127.0 (93.9–245.7)	242.3 (140.2–499.1)	*p* < 0.001
15–20 cpm	63.1 (47.4–86.5)	371.8 (217.0–602.6)	*p* < 0.001
20–30 cpm	44.1 (40.0–67.4)	128.0 (73.2–268.4)	*p* < 0.001
30–40 cpm	21.2 (13.6–28.5)	92.8 (48.8–180.8)	*p* < 0.001
40–50 cpm	11.5 (7.4–17.6)	29.8 (17.2–63.3)	*p* < 0.001
50–60 cpm	7.3 (3.9–15.3)	18.1 (9.4–35.3)	*p* < 0.001
(**b**)			
**AUC Frequency**	**Cohort 1**	**Cohort 3**	***p*-Value**
10–15 cpm	127.0 (93.9–245.7)	210.6 (135.7–374.6)	0.003
15–20 cpm	63.1 (47.4–86.5)	196.4 (73.0–436.8)	*p* < 0.001
20–30 cpm	44.1 (40.0–67.4)	90.8 (59.3–186.8)	*p* < 0.001
30–40 cpm	21.2 (13.6–28.5)	71.3 (27.5–125.5)	*p* < 0.001
40–50 cpm	11.5 (7.4–17.6)	22.5 (14.5–44.5)	*p* < 0.001
50–60 cpm	7.3 (3.9–15.3)	16.3 (7.5–38.4)	*p* < 0.001
(**c**)			
**AUC Frequency**	**Cohort 2**	**Cohort 3**	***p*-Value**
10–15 cpm	242.3 (140.2–499.1)	210.6 (135.7–374.6)	0.32
15–20 cpm	371.8 (217.0–602.6)	196.4 (73.0–436.8)	0.005
20–30 cpm	128.0 (73.2–268.4)	90.8 (59.3–186.8)	0.05
30–40 cpm	92.8 (48.8–180.8)	71.3 (27.5–125.5)	0.04
40–50 cpm	29.8 (17.2–63.3)	22.5 (14.5–44.5)	0.16
50–60 cpm	18.1 (9.4–35.3)	16.3 (7.5–38.4)	0.59

Area under the curve (AUC) calculated for each woman for a given frequency. Median AUCs were estimated and compared using the rank-sum test.

**Table 5:** (**a**). Linear regression analysis of GIMA biomarker AUC by frequencies for differences in non-endometriosis controls (Cohort 1) and subjects with endometriosis (Cohort 2). (**b**) Linear regression analysis of GIMA biomarker AUC by frequencies for differences in non-endometriosis controls (Cohort 1) and subjects with endometriosis (Cohort 3).

(**a**)				
**Frequency**	**Univariable Analysis**		Multivariable Analysis *	
	**Mean Difference (95% CI)**	***p*-Value**	**Mean Difference (95% CI)**	***p*-Value**
AUC_10–15_	227.4 (93.6–361.1)	0.001	278.3 (125.6–430.9)	0.001
AUC_15–20_	417.8 (321.0–514.7)	*p* < 0.001	402.4 (289.7–515.1)	*p* < 0.001
AUC_20–30_	147.9 (98.9–197.0)	*p* < 0.001	149.2 (93.2–205.3)	*p* < 0.001
AUC_30–40_	105.2 (77.6–132.8)	*p* < 0.001	102.1 (70.0–134.2)	*p* < 0.001
AUC_40–50_	31.9 (21.5–42.3)	*p* < 0.001	28.2 (16.6–39.9)	*p* < 0.001
AUC_50–60_	10.8 (2.3–19.4)	0.01	11.9 (2.2–21.7)	0.02
(**b**)				
**Frequency**	**Univariable Analysis**		Multivariable Analysis *	
	**Mean Difference (95% CI)**	***p*-Value**	**Mean Difference (95% CI)**	***p*-Value**
AUC_10–15_	85.4 (−30.4–201.2)	0.15	113.2 (−23.8–250.2)	0.11
AUC_15–20_	272.9 (160.5–385.3)	*p* < 0.001	302.0 (166.7–437.4)	*p* < 0.001
AUC_20–30_	88.6 (39.0–138.2)	0.001	95.5 (36.1–154.9)	0.72
AUC_30–40_	68.4 (40.5–96.2)	*p* < 0.001	71.2 (37.6–104.8)	*p* < 0.001
AUC_40–50_	23.4 (12.1–34.8)	*p* < 0.001	24.2 (10.7–37.7)	*p* < 0.001
AUC_50–60_	16.7 (5.8–27.6)	0.003	18.1 (5.6–30.7)	0.005

* Model adjusted for age, symptom score, water load, and BMI.

**Table 6:** Comparison of GIMA biomarker characteristics between subgroup 1 and subgroup 2 using median (IQR).

Frequency (Cpm = Cycles/Min)	Subgroup 2 Asymptomatic Non-Endometriosis Controls w/o Symptoms (n = 7)	Subgroup 1 Symptomatic Non-Endometriosis Controls (n = 55)	*p*-Value
10.0–15.0 cpm			
Baseline	5.8 (1.7–9.5)	5.1 (2.8–7.3)	0.9
10 min	3.0 (1.1–5.1)	4.8 (2.8–7.5)	0.2
20 min	2.0 (2.0–5.1)	4.0 (2.4–10.1)	0.1
30 min	4.4 (2.9–9.1)	4.7 (2.4–9.9)	0.9
15.0–20.0 cpm			
Baseline	2.4 (1.0–4.4)	2.0 (1.7–3.0)	0.8
10 min	0.8 (0.3–2.9)	2.1 (1.2–3.1)	0.1
20 min	1.2 (0.6–1.8)	1.8 (1.2–3.1)	0.3
30 min	2.1 (0.9–3.9)	2.0 (1.2–2.8)	*p* > 0.95
20.0–30.0 cpm			
Baseline	2.0 (1.0–2.4)	1.6 (1.0–2.8)	0.9
10 min	0.8 (0.4–1.9)	1.5 (0.9–1.9)	0.2
20 min	1.2 (0.5–1.5)	1.5 (0.9–2.0)	0.1
30 min	1.4 (1.3–2.6)	1.8 (0.9–2.3)	0.7
30.0–40.0 cpm			
Baseline	0.8 (0.2–1.6)	0.7 (0.5–1.2)	0.8
10 min	0.4 (0.1–0.9)	0.6 (0.4–0.9)	0.3
20 min	0.5 (0.4–0.9)	0.7 (0.4–0.9)	0.8
30 min	0.8 (0.4–1.1)	0.7 (0.4–1.1)	0.7
40.0–50.0 cpm			
Baseline	0.3 (0.1–0.7)	0.4 (0.2–0.6)	0.5
10 min	0.1 (0.1–0.5)	0.3 (0.2–0.5)	0.2
20 min	0.3 (0.2–0.5)	0.4 (0.2–0.6)	0.7
30 min	0.4 (0.2–1.0)	0.4 (0.2–0.6)	0.3
50.0–60.0 cpm			
Baseline	0.3 (0.2–1.1)	0.3 (0.1–0.4)	0.2
10 min	0.2 (0.04–1.4)	0.2 (0.1–0.4)	*p* > 0.95
20 min	0.4 (0.1–0.6)	0.2 (0.1–0.5)	0.9
30 min	0.4 (0.1–1.0)	0.2 (0.1–0.8)	0.7

Unadjusted median and interquartile ranges, calculated for each frequency ranging from 10 cpm to 60 cpm at times BL, 10, 20 and 30 min compared within Cohort 1 between subgroup 1 and subgroup 2 using rank-sum test.

**Table 7:** AI-derived permutations of prediction modeling for subjects aged ≤ 35. PPV is positive predictive value. NPV is negative predictive value.

Age ≤ 35 Years (N = 24)	Sensitivity	Specificity	PPV	NPV	C-Statistic	Correctly Classified
AUC_15–20_	76%	92%	89%	82%	91%	85%
AUC_15–20_ + Symptom Score	95%	96%	95%	96%	99%	95%
AUC_15–20_ + Symptom Score + Age	95%	96%	95%	96%	99%	95%
AUC_30–40_	71%	96%	94%	80%	88%	85%
AUC_30–40_ + Symptom Score	95%	92%	90%	96%	99%	93%
AUC_30–40_ + Symptom Score + Age	95%	92%	90%	96%	99%	93%
AUC_40–50_	52%	88%	79%	69%	79%	72%
AUC_40–50_ + Symptom Score	79%	92%	88%	85%	96%	86%
AUC_40–50_ + Symptom Score + Age	89%	92%	89%	92%	97%	91%
AUC_15–20_ + AUC_30–40_ + Symptom Score	95%	96%	95%	96%	99%	95%
**AUC_30–40_ + AUC_40–50_ + Symptom Score**	**95%**	**96%**	**95%**	**96%**	**>99%**	**95%**
AUC_15–20_ + AUC_30–40_ + AUC_40–50_ + Symptom Score	95%	96%	95%	96%	>99%	95%

The yellow-highlighted bold values designate the best predictive model for subjects aged 35 and younger, in Cohort 2.

**Table 8:** AI-derived permutations of prediction modeling for subjects aged ≥36.

Age ≥ 36 Years (N = 19)	Sensitivity	Specificity	PPV	NPV	C-Statistic	Correctly Classified
AUC_15–20_	61%	95%	88%	80%	83%	82%
AUC_15–20_ + Symptom Score	70%	92%	84%	83%	90%	83%
AUC_15–20_ + Symptom Score + Age	78%	97%	95%	88%	94%	90%
AUC_30–40_	70%	95%	89%	83%	89%	85%
AUC_30–40_ + Symptom Score	83%	92%	86%	89%	97%	88%
AUC_30–40_ + Symptom Score + Age	91%	95%	91%	95%	99%	93%
AUC_40–50_	48%	89%	73%	73%	81%	73%
AUC_40–50_ + Symptom Score	78%	89%	82%	87%	92%	85%
AUC_40–50_ + Symptom Score + Age	91%	92%	88%	94%	95%	92%
AUC_15–20_ + AUC_30–40_ + Symptom Score	91%	95%	91%	95%	99%	93%
**AUC_30–40_ + AUC_40–50_ + Symptom Score**	**91%**	**95%**	**91%**	**95%**	**98%**	**93%**
AUC_15–20_ + AUC_30–40_ + AUC_40–50_ + Symptom Score	91%	95%	91%	95%	98%	93%

The yellow highlighted bold values designate the best predictive model for subjects aged 36 and older, in Cohort 2.

**Table 9:** GIMA biomarker performance in predictive model on endometriosis-positive validation cohort segregated by age subgroups age ≤ 35 and ≥36 (Cohort 3).

Age-Derived Subsets (N = 47)	Sensitivity	Specificity	PPV	NPV	C-Statistic	Correctly Classified
Age ≤ 35 years (N = 25)	86%	96%	86%	96%	91%	91%
Age ≥ 36 years (N = 22)	84%	95%	84%	95%	90%	91%

## Data Availability

Data are available from the Endometriosis and Neuroenterology Institute, marknoar@gmail.com.

## Supplementary Materials

The following supporting information can be downloaded at: https://www.mdpi.com/article/10.3390/jcm13102866/s1, Figure S1A–F: Distribution of AUC for frequencies 10–60 cpm for controls (no endometriosis) and women with endometriosis.
