# Supplementary material for: Gastrointestinal Myoelectrical Activity (GIMA) Biomarker for Noninvasive Diagnosis of Endometriosis
Source: J Clin Med. 2024 May 13;13(10):2866. doi: 10.3390/jcm13102866 (PMC11122642; doi:10.3390/jcm13102866)
Supplement: Supplementary file 1 [file jcm-13-02866-s001.zip › jcm-2977359-supplementary.pdf]

Figure S1 A-F: Distribution of AUC for frequencies 10 – 60 cpm for controls (controls) and women with endometriosis (cases)

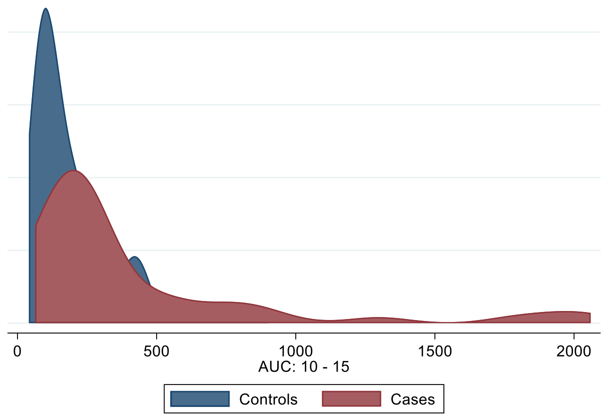

A. Comparison of AUC difference at 10 -15cpm between symptomatic or asymptomatic healthy controls (Controls) and women with endometriosis (cases) using kernel density plots.

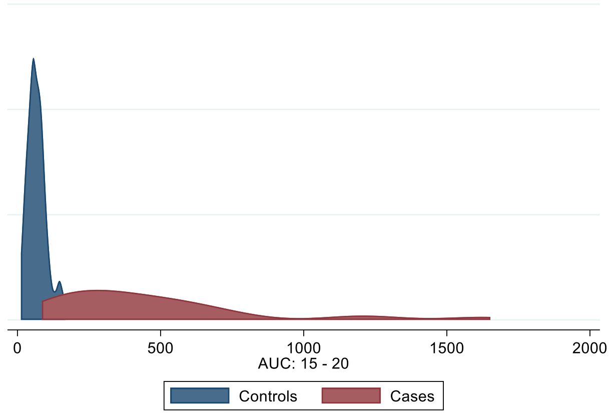

B. Comparison of AUC difference at 15 -20 cpm between symptomatic or asymptomatic healthy controls (Controls) and women with endometriosis (cases) using kernel density plots

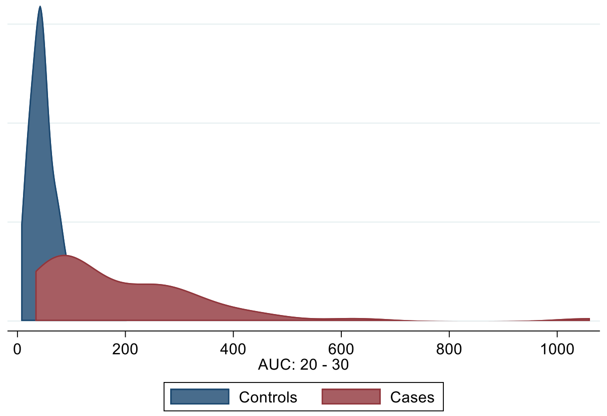

C. Comparison of AUC difference at 20 -30 cpm between symptomatic or asymptomatic healthy controls (Controls) and women with endometriosis (cases) using kernel density plots.

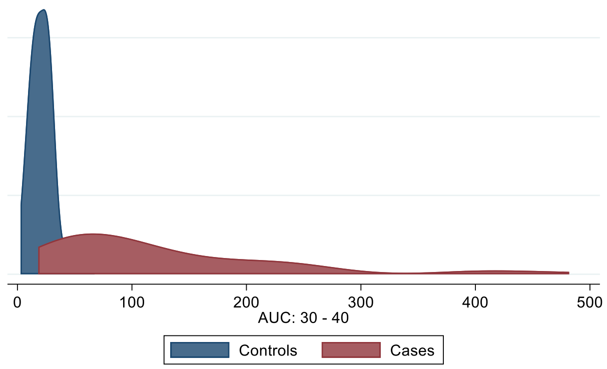

D. Comparison of AUC difference at 30 -40 cpm between symptomatic or asymptomatic healthy controls (Controls) and women with endometriosis (cases) using kernel density plots.

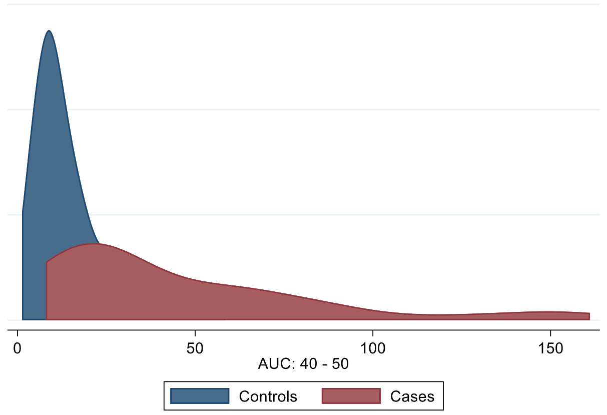

E. Comparison of AUC difference at 40 -50 cpm between symptomatic or asymptomatic healthy controls (Controls) and women with endometriosis (cases) using kernel density plots.

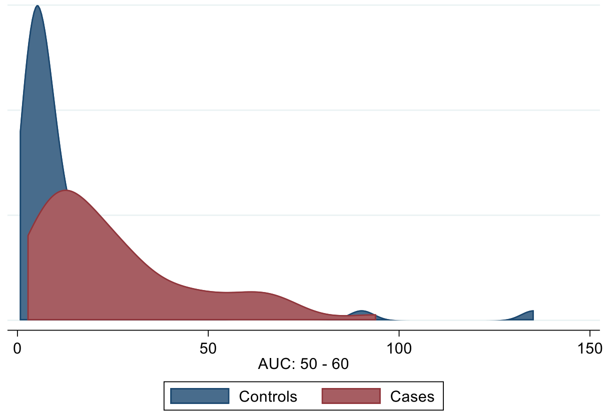

F. Comparison of AUC difference at 50 -60 cpm between symptomatic or asymptomatic healthy controls (Controls) and women with endometriosis (cases) using kernel density plots.
